# Rigor and Reproducibility in Electrocatalysis: Integrating Theory and Experiment

**DOI:** 10.1021/jacs.6c09028

**Published:** 2026-10-28

**Authors:** Líney Árnadóttir, Alexander Bagger, Jason S. Bates, Ezra L. Clark, Guoxiang Hu, Michael J. Janik, Nirala Singh, Eric Stuve, G. T. Kasun Kalhara Gunasooriya, Robert E. Warburton

**Affiliations:** † School of Chemical, Bioengineering, and Environmental Engineering, Oregon State University, Corvallis, Oregon 97330, United States; ‡ Physical and Computational Sciences Directorate and Institute for Integrated Catalysis, Pacific Northwest National Laboratory, Richland, Washington 99354, United States; § Department of Physics, Technical University of Denmark, Kongens Lyngby 2800, Denmark; ∥ Department of Chemical Engineering, 2358 University of Virginia, Charlottesville, Virginia 22903, United States; ⊥ Department of Chemistry, University of Virginia, Charlottesville, Virginia 22903, United States; # Department of Chemical Engineering, 8082 The Pennsylvania State University, University Park, Pennsylvania 16802, United States; ∇ School of Materials Science and Engineering, 1372 Georgia Institute of Technology, Atlanta, Georgia 30332, United States; ○ Department of Chemical Engineering, University of Michigan, Ann Arbor, Michigan 48109-2136, United States; ◆ Department of Chemical Engineering, University of Washington, Seattle, Washington 98195, United States; ¶ School of Sustainable Chemical, Biological and Materials Engineering, 6187 University of Oklahoma, Norman, Oklahoma 73019, United States; △ Department of Chemical and Biomolecular Engineering, 2546 Case Western Reserve University, Cleveland, Ohio 44106, United States

## Abstract

Electrocatalysis is central to electrified energy conversion technologies, including the production of fuels and chemicals from renewable electricity. In recent years, atomistic modeling based on density functional theory (DFT) has become an indispensable tool for catalysis researchers because it can provide a molecular-level perspective of catalytic reactivity that is otherwise inaccessible through experimental measurements. Computational electrocatalysis model inputs are intended to capture fundamental understanding of the complex interfacial environment of the catalytic active site, but they rely on practical approximations that can affect the reliability of insights gained from DFT. This Perspective outlines a framework for rigorous and reproducible integration of theory and experiment in heterogeneous electrocatalysis research. We discuss state-of-the-art computational methods and sources of error in model development and interpretation. We describe opportunities for validation of theoretical models and experimental interpretations as they pertain to the structure of active sites, reaction energetics, and microkinetic modeling. We provide examples to help computational researchers develop models that move beyond obtaining qualitative agreement with reactivity trends, and toward the development of rigorous mechanistic insights that can be leveraged to predict promising new catalysts or operating conditions. Rigor and reproducibility are enhanced when theory and experiment have multiple nodes of connection, thereby enhancing the longevity and robustness of conclusions and insights about electrocatalytic reactivity.

## Graphical abstract

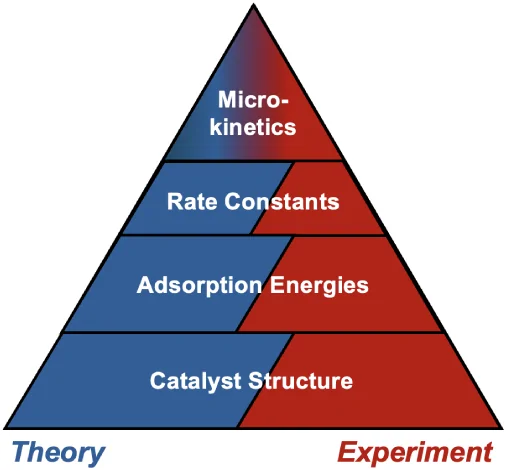

## Introduction

Electrocatalysis is foundational to clean energy conversion, enabling a range of sustainable chemical transformations that are central for advancing next-generation energy technologies.^1^ Unlike gas-phase heterogeneous catalysis, where well-defined ultrahigh vacuum conditions enable direct comparisons between theory and experiment, electrocatalysis operates at complex solid−electrolyte interfaces where the active site comprises not only the catalyst surface atoms but also the surrounding solvent and electrolyte. Moreover, the aggregate dynamical structure of the electrode–electrolyte interface is influenced by the applied potential and local electric fields.^2–4^ Atomistic simulations aim to add molecular-level insights into catalyst structure, dynamics, and reactivity, but models often rely on inputs and initial conditions that are poorly defined when compared to the complex behavior of electrochemical interfaces. This complexity has led to a proliferation of “decorative DFT” studies that predict trends and mechanisms without systematic experimental validation, and conversely, experimental work that proceeds without leveraging the mechanistic insights available from theory.^5^ The result is a literature rich in qualitative insights but often lacking the quantitative agreement necessary to confidently predict new catalyst materials or operating conditions.

This Perspective provides a framework for achieving rigor and reproducibility in combined theoretical-experimental electrocatalysis studies. The challenges that affect experimental measurements^6–12^ and theoretical calculations^2–4,13–17^ in electrocatalysis are described in other essential perspectives and reviews. The objective of this Perspective is to provide both new and experienced theoretical and experimental electrocatalysis practitioners with successful examples of theory/experiment integration. We furthermore aim to identify emerging opportunities to establish more robust theoretical and experimental protocols for mechanistic understanding in electrocatalysis that may be enabled through integrated theory and experiment. Herein, we focus on the *integration of theory and experiment*, and strategies for internal validation of both theoretical models and experimental interpretation through four interconnected topics: catalyst structure and active site identification, adsorption energies under electrochemical conditions, activation energies and transition states, and microkinetic modeling. For each topic, we discuss the current state of the art in both computational methods and experimental measurements, identify key sources of error and uncertainty, and provide pragmatic recommendations for establishing robust comparisons between theory and experiments. We encourage the readers to explore complementary perspectives on electrochemical kinetics,^18^ *operando* characterization,^10^ and active site quantification^12^ for a more in-depth understanding of achieving rigor and reproducibility in these topics. Our emphasis throughout is on moving beyond qualitative agreement toward quantitative benchmarks, recognizing that while perfect accuracy remains elusive, systematic approaches to uncertainty quantification and multiobservable validation can dramatically improve the predictive power of electrocatalysis research.

## Catalyst Structure

The active site of a catalyst dictates the stability of reaction intermediates and transition states, how fast the reaction proceeds, and which products are formed. Thus, a well-characterized and well-defined active site establishes a necessary foundation for rigorous integration of theory and experiment (Figure 1). Electrocatalysis occurs at an electrically polarized solid-electrolyte interface, and therefore the active site is comprised of both the atomic arrangements of the catalyst surface (e.g., metal terraces, steps, or corner atoms) and the solvent and electrolyte.^11^ Rigorous and reproducible comparisons of theory and experiments therefore require comprehensive efforts to identify the active sites. These efforts include characterizing the active site under reaction conditions, synthesizing well-defined catalysts, or analyzing systematic trends in activity and selectivity with descriptors. Simulations and experiments can be linked either through a well-characterized active site or through preparation of well-defined electrode surfaces, such as single crystals^19,20^ or heterogenized molecular catalysts.^21–23^ If characterization or well-defined surfaces cannot be used, trend-based comparisons across a diverse array of catalyst materials may be established.

**Figure 1:**
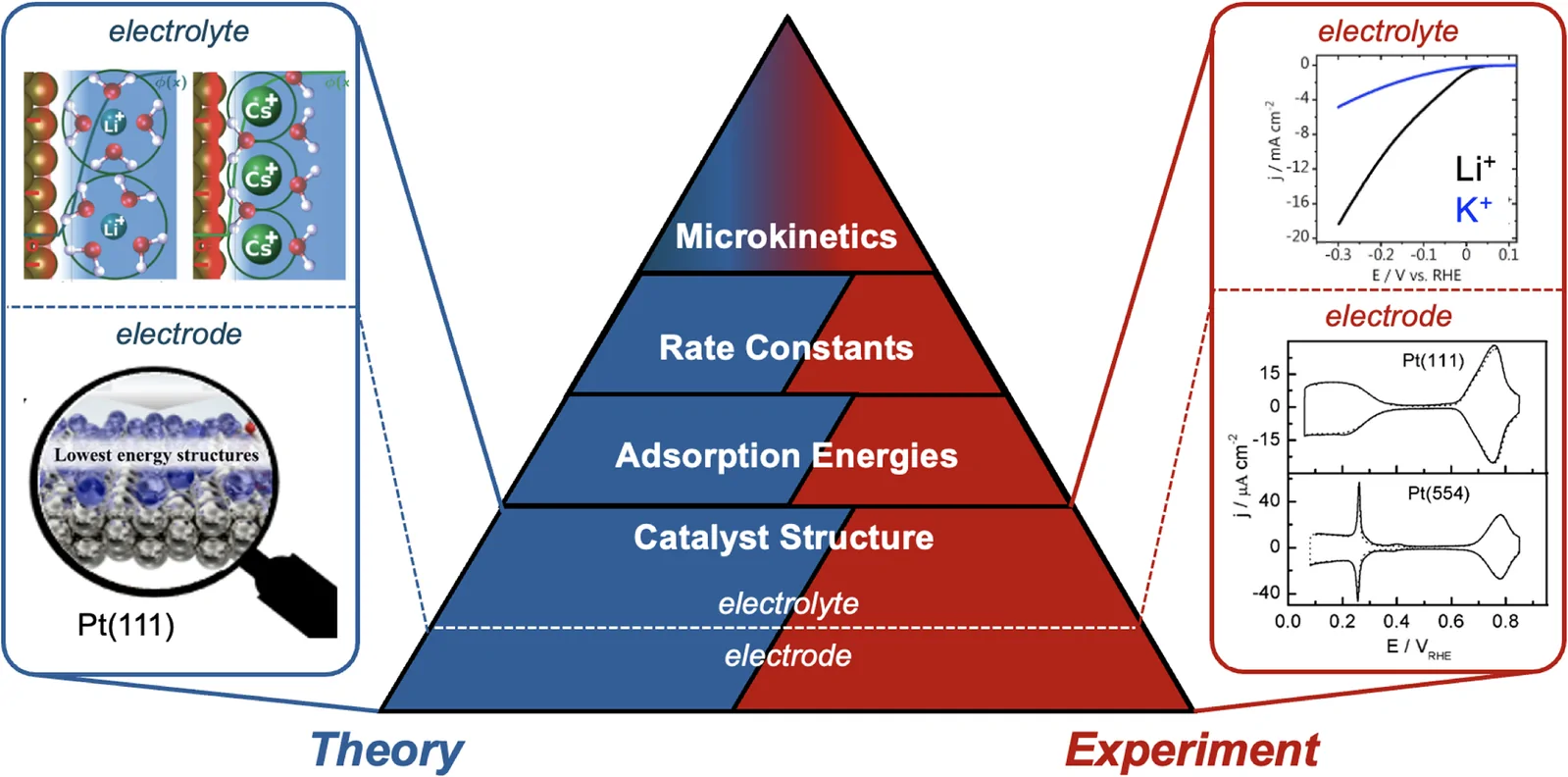
Rigorous scientific insights can be obtained between theory and experiment through a strong foundational understanding of the catalyst structure, which is influenced by the properties of both the electrode and electrolyte sides of the electrified solid–liquid interface. Theory and experiment can each be used to provide complementary insights into the effects of the electrode and electrolyte on catalyst structure and reactivity. Model inputs pertaining to electrode and electrolyte structure influence the quantitative and qualitative insights that can be gained from adsorption energy and rate constant calculations. Errors and uncertainty in these calculated quantities propagate to uncertainty in experimentally measurable quantitates that can be derived from microkinetic modeling. Theory panels are reproduced with permission from (top left) ref. 35, copyright 2019 Royal Society of Chemistry; and (bottom left) ref. 33, copyright 2020 American Chemical Society. Experiment panels are reproduced with permission from (top right) ref. 36, copyright 2021 American Chemical Society; and (bottom right) ref. 37, copyright 2008 Royal Society of Chemistry.

Experiments can define active site structures through detailed characterizations or using well-defined catalysts. However, atomistic characterization of the active site under operating conditions is difficult, and the stability and dynamics of catalyst structure should be considered rather than assuming a static structure. Detailed characterizations can be performed *in situ* or *operando*, and readers interested in this topic are encouraged to refer to a related perspective on this topic.^10^ Characterization methods can be directly compared with simulations as long as the surface structure remains stable under reaction conditions. For example, electrochemical scanning tunneling microscopy^24,25^ has been used to quantify edge sites on MoS_2_, and show that they are active for H_2_ evolution because the rate scales with the number of edge sites.^26^ Experiments and simulations of well-defined single crystal electrodes are another exemplary approach to achieve an accurate active site comparison between theory and experiment.^27–29^ Cyclic voltammograms (CVs) of single crystal electrodes can identify electrosorption onto specific facets.^19,30^ For example, CVs on Pt(111) show different features related to adsorption of electrolyte anions and other surface species (e.g., H*, OH*) when the electrolyte is varied. The H* and OH* regions of the CV of Pt(111) in HClO_4_ can be related to DFT structures from *ab initio* phase diagrams or by analysis of potential-dependent adsorption rates.^31–34^ In this manner, atomistic structures can be identified through the fingerprinting of a CV and corroborated by theory, providing direct insight into the interfacial structure.

Simulations provide tools to predict the stable active site configurations under reaction conditions using thermodynamic reference schemes. As an initial prediction, Pourbaix diagrams are used to find the stable crystal structure under reaction conditions.^38–40^ Following this, surface energies can be used to find the crystal facet that is primarily exposed; however, this does not guarantee that the most active facet is the most exposed one. The surface coverage of species on the active site can then be obtained using reference schemes, e.g., the computational hydrogen electrode (CHE)^41^ for hydrogen or hydroxyl coverages, though other anionic or cationic species can be referenced. Together, this thermodynamic framework enables the construction of surface Pourbaix diagrams, which map the most stable surface structures and adsorbate coverages as a function of potential and pH. In addition to surface coverages, surface reconstructions and defect formation under reaction conditions may also play an important role, as demonstrated by *operando* studies revealing potential-driven, reversible restructuring of oxide catalyst surfaces.^42–45^ These structures can be directly compared with CV measurements^46^ or *operando* spectroscopy,^10^ although the complexities of adsorbate–adsorbate interactions and diversity of binding sites must be considered.^46–49^ Addressing the thermodynamics of the active site is essential to avoid the common misconception that the initial catalyst submerged in an electrolyte under a specific potential is identical to the working electrocatalyst. Examples of this include NiFe for oxygen evolution reaction which transitions into NiOOH under reaction conditions^50–52^ or Cu oxides which get reduced at CO_2_ reduction conditions,^53–55^ as the reduction is carried out far below the standard reduction potential of Cu oxide.^56,57^ Additionally, surface facets can reconstruct in response to the applied potentials and interactions with the electrolyte. For example, simulations indicate that Pt(111) may reconstruct to Pt(100) because strong hydrogen adsorption on Pt(100) introduces a driving force for a facet change.^58^ Strong-binding adsorbates can also cause surface segregation in alloys^59^ and drive the migration of surface atoms into catalytically active nanoclusters.^60^ In some cases, metastable phases can further complicate predictions based on thermodynamic stability alone.^61,62^ Computations and experiments can work in concert to identify active site structures and surface coverages starting from simple models and building in complexity as needed to describe shared observables.

Electrocatalytic active sites are distinct from those in gas-phase catalysis because the electrolyte is an integral part of the active site. The electrolyte itself can modify the active sites, further complicating understanding of the electrode–electrolyte interface. Computationally, specifically adsorbed reaction intermediates (e.g., H*, OH*) and electrolyte ions can be modeled well, but nonadsorbing electrolyte species are far more challenging to describe. When an electrode is submerged in an electrolyte under an applied potential, it is challenging to model how ions from the solution arrange themselves near the surface to screen the electrode’s charge. Despite these challenges, nonspecific ion interactions are important to consider computationally, especially when they affect catalytic reactivity such as in the case of the hydrogen evolution reaction on Au and Pt in electrolytes containing Li^+^ or K^+^ (Figure 1).^36^ Electrolytes can be described theoretically using either implicit or explicit electrolyte models.^16,63,64^ Explicit electrolyte models require knowledge about the surface structure and the electrolyte composition in the electric double layer (EDL). Some implicit modeling approaches provide predictions of near-surface electrolyte species concentrations;^65–67^ however, such models are limited in their treatment of chemical interactions via either polarizable continuum or classical force field models.^68,69^ The electrolyte is a critical component of the active site that warrants care in model development because of the sensitivity of energetics to the choice of EDL model.^70,71^ DFT-based *ab initio* molecular dynamics (AIMD) simulations can be used to model the electrolyte explicitly, but are limited to picosecond time scales. Recent developments in machine learning interatomic potentials may mitigate time scale challenges associated with these simulations while maintaining DFT-level accuracy within their training domain.^72–74^ However, MLIP transferability to electrified interfaces outside the training domain remains limited, with performance degrading substantially on out-of-domain configurations. Rigorous uncertainty quantification and active learning are therefore necessary before these potentials can be applied with confidence to unexplored interfacial configurations. More specific details about modeling the EDL will be included in the sections below in their appropriate context.

In summary, any combined experimental–theoretical study necessarily begins with an iterative process of proposing and distinguishing between candidate active site structures. It is important to recognize that different catalyst classes intrinsically show different extents of molecular precision and diversity in their active site structures.^11^ In rare cases, the molecular structure of active sites can be experimentally determined; here, theory can directly describe and predict experimentally inaccessible properties like electronic structure. As active site complexity increases at the expense of molecular precision, theory becomes indispensable for active site identification. In this pursuit, experiments and theory must build a suite of shared observables both structural and catalytic and engage iteratively to eliminate candidate active site structures. Adsorption thermodynamics and chemical kinetics are chief among these shared observables, as discussed below.

## Adsorption Energies

Adsorption energies influence macroscopic reaction rates by controlling adsorption rates, desorption rates, and coverages, making accurate modeling of adsorption energies critical for meaningful electrocatalytic comparisons between theory and experiment. Methods to compare adsorption energies to experiment, without needing to consider the additional effects of kinetics (e.g., activation barriers), are an important stepping stone for rigor in the integration of theory and experiment in electrocatalysis. Gas-phase calculations and comparisons to gas-phase experiments are an excellent starting point toward understanding coverage effects such as adsorbate–adsorbate interactions. Furthermore, benchmark repositories have established comparisons between gas-phase adsorption energy across different DFT functionals and adsorption calorimetry experiments.^75^ Modeling and experiment comparison of electrocatalytically relevant adsorption energies have the same challenges as gas-phase adsorption energies, but there are additional important effects for electrocatalysis. In this section we highlight some of these effects, particularly solvation and solvent displacement and potential/field effects. We then describe techniques to model these effects and techniques to improve rigorous comparisons between experiment and theory.

The role of solvent in adsorption can be particularly significant in electrocatalysis. One contributing effect to adsorption is the solvation of the adsorbing molecule itself, and how the energetics change when the molecule goes from being fully solvated to adsorbed on a solid electrode surface in a solvent. Another significant effect, as Gileadi and others have shown, is that electrosorption is a replacement reaction, meaning solvent at the electrode surface must be displaced upon adsorption of a different molecule.^76^ This is especially important for larger molecules (e.g., aromatics or longer chain hydrocarbons) which entail a non-negligible enthalpic “penalty” for the displacement of multiple solvent molecules from the electrode surface. Electrocatalytic adsorption, where the solvent is displaced, also has interesting consequences on entropy. In the gas-phase, adsorption is entropically unfavored, so species desorb at higher temperatures, while in liquid-phase, solvent displacement can make adsorption less entropically unfavored, or even entropically favored. This would occur when the entropy loss of the adsorbing molecule is offset by the entropy gain of the solvent displacement. This difference in entropy can have important consequences for the temperature dependence of adsorption.^76–78^ The solvent displacement associated with adsorption can also affect the potential dependence of adsorption, even for uncharged adsorbates, in solvents where the potential or field influences the solvent-electrocatalyst interaction.^79^

Potential has very clear effects on adsorption on catalyst surfaces and is commonly studied using cyclic voltammetry (CV). The CVs of Pt(111) in Figure 1 show the hydrogen underpotential deposition region where the favorable (negative free energy) adsorption of H onto Pt causes an adsorption event at more positive potentials than the reversible hydrogen electrode.^28^ Because this adsorption involves an electron transfer (H^+^ being reduced to an adsorbed H atom, H*) we measure reduction current around 0.1 to 0.3 V vs RHE and a corresponding oxidation current associated with the desorption of that adsorbed hydrogen in the reverse (forward) sweep. The experimental cyclic voltammogram can be compared to a simulated cyclic voltammogram generated by the potential-dependent adsorption energies from computational models. An example on a Pt(553) electrode is shown in Figure 2A, where CVs in acid and in alkaline conditions are compared to understand how pH influences the adsorption feature in the hydrogen underpotential deposition region. An important caveat when comparing experiment to theory for understanding adsorption is ensuring that the experimental CV is conducted under conditions where no reactions are occurring other than adsorption. Furthermore, species characterized under noncatalytic conditions may be spectators rather than catalytically relevant surface species: a classic example is underpotential-deposited H species are spectators during the hydrogen evolution reaction.^80^ In general, steady-state spectroscopic measurements of surface coverage, even under catalytic conditions, still may be convoluted by spectator species.^81^

**Figure 2:**
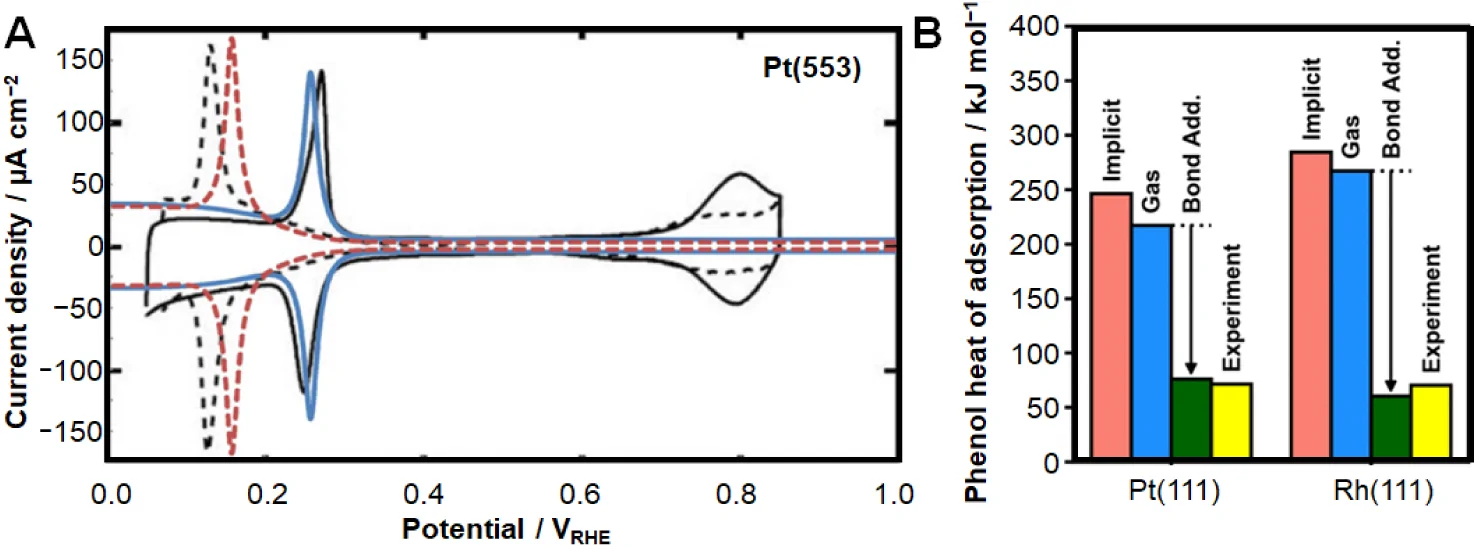
**A.** Experimental cyclic voltammogram (CV) of Pt(553) measured in 0.1 M HClO_4_ (dashed black line) and in 0.05 M NaOH (solid black line) from ref. 82 and computational CV simulated at 50 mV/s in acid (free from coadsorbed alkali, red dashed line) and base (with coadsorbed alkali, blue solid line). Reproduced with permission from ref. 83, copyright 2016 Wiley-VCH. **B.** Heat of adsorption of phenol onto Pt(111) and Rh(111). Includes gas phase adsorption energy calculated by DFT and validated for Pt by calorimetry (“Gas”), experimental aqueous phase adsorption energy extracted through adsorption isotherms (“Experiment”), and simulations for aqueous using implicit solvent (Implicit) and through bond-additivity model (“Bond Add.”). Reproduced with permission from ref. 84, copyright 2020 American Chemical Society.

Modeling approaches of potential-dependent adsorption often begin with the CHE model,^41^ but numerous additions are needed for improved accuracy. Within the CHE model, adsorption energies are calculated using DFT and the potential dependence is incorporated using the thermodynamic relationship between electrode potential and chemical potential of ions. The CHE model uses gas-phase calculations and reference redox reactions to determine the net chemical potential of ions and their stoichiometric electron equivalents, such that the associated electrochemical adsorption energies are computed on the basis of ion activities rather than concentration. The CHE has been used to study potential-dependent surface processes like H^+^ transfer to surfaces or adsorbed intermediates,^41^ chloride ion adsorption,^85^ nitrate,^86^ and others through gas-phase calculations, and is amenable to the inclusion of implicit or explicit solvation effects. Yet the CHE is limited in that it does not explicitly describe electrification of the interface or the redistribution of charge in the electrode and electrolyte that accompanies changes in potential. Thus, thermodynamic modeling of adsorption using the CHE often assumes integer and stoichiometric electron transfer in electrosorption, and ignores partial electron transfer from the displaced solvent. Making this more challenging, many of these effects are coupled. Adsorption at solid–liquid interfaces requires displacement of the solvent, and the energetics of both the adsorbate and the solvent displacement may depend on the applied potential.

To address the complexity of electrochemical interfaces, there has been significant effort in the development of computational methods to accurately account for surface solvation, solvent displacement, and electrification in the calculation of adsorption energies and barriers that incorporate accurate treatment of solvation and solvent. For example, the simulated CV of Pt in Figure 2A incorporated explicit water molecules during the simulation to consider the effect of surface solvation on adsorption. The electrolyte can be modeled using implicit^16,87–89^ or explicit^64^ solvation models, or hybrid approaches combining both. Implicit solvation methods capture long-range dielectric screening efficiently but may struggle with hydrogen bonding, solvent structuring, and specific ion effects. Explicit water layers provide more realistic interfacial configurations but are limited by sampling and computational cost. Hybrid solvation methods partially address these limitations but remain sensitive to how dielectric properties, cavity definitions, and counter-charge distributions within the electric double layer are parametrized. Some implicit solvent models address limitations of radial- or density-based implicit solvation via nonlocal cavity formation or classical density-based representations of implicit electrolytes.^90–95^ Importantly, results from implicit solvent models are sensitive to the parametrization of key electrolyte properties such as dielectric constant, as well as the distribution of counter-charges within the EDL.^16,65,70^ The choice of exchange-correlation functional further affects predicted adsorption strengths because water structure and interfacial polarization are highly functional-dependent. Recent theoretical frameworks directly quantify the free-energy cost of solvent displacement upon adsorption, enabling systematic corrections for the removal of interfacial water.^78^ An example considering the interactions of displaced solvent is shown in Figure 2B, where the penalty of displacing a water solvent is estimated for phenol adsorption on Pt(111) and Rh(111).^69,84^ This correction is found to more closely match the experimental adsorption energy than when using implicit water.

Accurately modeling electrification of the interface and the effect of the potential on the redistribution of charge in the electrode and electrolyte is especially important when adsorption energies or transition states are strongly influenced by local electric fields or solvent reorganization. Changes in applied potential can also alter the oxidation state (valence) of surface atoms and adsorbates, which directly affects adsorption energetics and reaction pathways.^96,97^ For example, the electrosorption valency (i.e., the number of transferred electrons required to maintain a constant potential) of halides and other anions can be potential and coverage dependent, which influences the competition for surface sites between ions and reactive intermediates in catalysis.^96,98–102^ The effects of applied potential can be evaluated using analytical corrections,^70,103–105^ grand-canonical DFT (GC-DFT),^106–111^ or other open boundary condition approaches,^112–116^ to model reaction energetics and activation barriers at constant potential. Extrapolation and analytical corrections incorporate energetic contributions from finite-cell effects and from capacitive and field–dipole interactions.^70,103–105,117^ In contrast, GC-DFT calculations are performed at constant potential where and excess charge is typically compensated by a counter-charge distribution.^106,107,111^ GC-DFT methods address the challenge of controlling the applied potential and surface charge; however, any shortcomings underlying the choice of solvation treatment remain.

Ultimately, comparison between simulation and experiment for adsorption is dependent on rigorously obtained experimental adsorption isotherms or adsorption energies for benchmarking first-principles calculations. Benchmarking of gas-phase surface reaction energetics suggests typical functional errors of ∼0.1−0.2 eV.^75^ Charged ion solvation energy errors may be as large as 0.1 eV, although errors may be smaller for neutral molecules.^16,118,119^ DFT practitioners should expect to inherit these sources of error and uncertainty, in addition to uncertainty regarding the solvent properties at the interface compared to bulk solution. First-principles calculations can be calibrated through comparison to experiment. Cyclic voltammetry is a commonly used experimental technique but only contains information on charge transfer (current), and thus it is difficult to determine what species is undergoing charge transfer, and CV does not give direct information on noncharge transfer events. Therefore, it should be supplemented with other techniques that study adsorption or desorption. One technique is to measure desorption of adsorbed species as a function of potential by varying the electrode potential and using electrochemical mass spectrometry to quantify what species desorb from the electrode surface into the bulk electrolyte.^120,121^ Electrochemical mass spectrometry has the advantage of being able to quantify the species desorbing based on their mass fragments. Quartz crystal microbalances can also study adsorption by detecting change in the mass of the electrode due to adsorbing or desorbing species, but this does not directly identify the composition of those species, only their mass.^120^ Quartz crystal microbalances are another method to study the extent of adsorption based on the change in electrode mass associated with adsorption.^120^ Thin-layer coulometry, where a very small electrolyte volume is put in contact with an electrode, is also a method to extract adsorption coverages on electrode surfaces.^122^ Typically, the number of adsorbing molecules onto a planar electrode is not large enough to significantly change the bulk electrolyte concentration. However, in thin-layer coulometry the high electrode surface to electrolyte volume ratio allows bulk changes in the concentration of adsorbate to be quantified, and thus the amount of adsorbate (and therefore coverage) can be quantified by these bulk concentration changes. Aside from adsorption isotherms, calorimetry is an important technique to benchmark gas phase calculations,^75^ but also has the potential to help with electrocatalysis through coadsorption.^123,124^ Microcalorimetry measurement of solvent-surface interactions can be used as benchmarks for computations,^75,125–127^ but solvent-surface interactions also contribute to adsorption energy in liquid because of the significant energy cost of replacing the solvent molecules.^69,128,129^ Microcalorimetry can be used to measure the effect of coadsorbates as a benchmark for calculations as a first step toward addressing explicit coverage and solvent effects. Liquid phase calorimetry is useful for understanding adsorption energetics of organic molecules.^69,124^ We emphasize that reversibility is important for rigorously understanding adsorption as opposed to decomposition of a species. For example, if a species decomposes reversibly upon adsorption, its energy or coverage cannot be compared to the computed free energy of the molecule without decomposition. Therefore, we encourage researchers to investigate hysteresis of adsorption isotherms or, if possible, characterize the structure of the adsorbed species.

## Activation Energies and Rate Constant Theories

The rate constants of elementary steps affect overall rates and selectivities of electrocatalytic reactions. First-principles rate constant modeling requires a rate constant theory (e.g., transition-state theory or Marcus theory) with parameters that can be derived using computational chemistry. Common rate constant models used in heterogeneous electrocatalysis generally express the rate constant *k* using an Arrhenius form: $k=A\exp\left(-\frac{E_{\mathrm{act}}}{k_{B}T}\right)$ , where *A* is the pre-exponential factor, *E*_act_ is the activation energy (enthalpy), *k*_B_ is the Boltzmann constant, and *T* is the temperature. The pre-exponential factor *A* is often implicitly assumed to entail a classical attempt frequency and is often approximated as $A=\frac{k_{B}T}{h}\exp\left(\frac{\Delta S^{‡}}{k_{B}}\right)c^{o}$ , where *h* is Planck’s constant, ΔS^‡^ is the activation entropy, and *c* ^o^ is a standard reference concentration (or pressure for gas-phase reactants). We note that this expression neglects the contributions of solvent reorganization energy, solvent relaxation dynamics, tunneling probabilities, Franck–Condon factors, and electronic density of states to the pre-exponential factor.^130–134^ For further details, we direct interested readers to more extensive reports on these topics.^17,135–137^ Transition-state theory models of elementary rate constants from computational chemistry require an atomistic model that can be used to estimate activation energies. Any atomistic model must make assumptions about the structure of the active site associated with an elementary step, which includes both the atom(s) on the catalyst surface that bind intermediates and transition states as well as the structure and dynamics of the surrounding electrolyte, as described in the “Adsorption Energies” section. Faradaic elementary steps involve charged intermediates, the energetics of which are sensitive to electrode surface charge, solvent structure, and local electric fields. These sensitivities can lead to substantial deviations between the activation energies predicted by computation and those inferred from experiments, particularly when subtle changes in interfacial structure produce nontrivial shifts in local electric fields. Such deviations could manifest as potential-dependent symmetry factors for specific elementary steps.^18,138–140^

It is important to note that experimentally, elementary-step activation barriers are rarely accessible to measurements. Catalytic currents reflect a net forward rate of a catalytic cycle comprised of multiple elementary steps under specific conditions. Thus, activation barriers obtained from Arrhenius analysis at constant potential *E* on a stated reference scale are referred to as apparent activation energies $\left(E_{\mathrm{app}}=RT^{2}{\left(\partial \ln i/\partial T\right)}_{E}\right)$ . The apparent activation energy can change as a function of surface coverage and the kinetic relevance^139,141^ and reversibilities^142^ of elementary steps, all of which can be a strong function of applied potential.^18^ Therefore, we caution against direct comparison of apparent activation energies with theoretical transition-state calculations without detailed mechanistic analysis, as discussed below in the *Microkinetic Modeling and Kinetic Insights* section.

The challenges associated with accurate adsorption energy calculations become even more complex when computing energetics for transition states. As with adsorption energies, activation energies can be computed within either the constant-charge or constant-potential (electronically grand-canonical) ensemble. Neither approach is fool-proof, however, as modeling within either ensemble has associated limitations.^13^ Periodic DFT calculations of activation barriers performed at a constant zero total charge suffer from changes in the work function (electrode potential) along the reaction coordinate (Figure 3A). GC-DFT methods enable activation energy calculations at constant potential by compensating the net charge of the atomic system by countercharge in the implicit solvent (Figure 3B).^106,107,109,114^ However, GC-DFT calculations rely on approximate models for local solvation, which may include implicit solvent models that require assumptions about EDL properties (e.g., dielectric constant, EDL width, and counter-charge distribution). With equivalent parametrization of EDL parameters, analytical grand-canonical DFT (aGC-DFT) methods^70^ reproduce activation barriers computed using explicit GC-DFT.^71,143,144^ As shown in Figure 3B uncertainty in EDL parameters translates to high uncertainty in activation free energies when there is a significant change in the dipole moment along the reaction coordinate.^71^ While GC-DFT approaches provide one solution to the problem of controlling the interfacial potential in computational electrocatalysis, they do not eliminate all potential sources of error in activation free energy calculations. More fundamentally, it is unclear whether maintaining a constant electrode potential along the reaction coordinate of an activated reaction is appropriate given that equilibration with a potentiostat is much slower than local solvent reorganization and double layer relaxation.^145^ Yet, solvent reorganization is commonly viewed as a thermally activated process that for large enough barriers may occur on similar time scales to those of potentiostat equilibration.^146^ Some estimates suggest that the electrode potential should be equilibrated along the reaction coordinate for reactions with barriers larger than ∼0.35 eV, justifying the validity of the grand-canonical ensemble along the reaction coordinate of an elementary step.^137^

**Figure 3:**
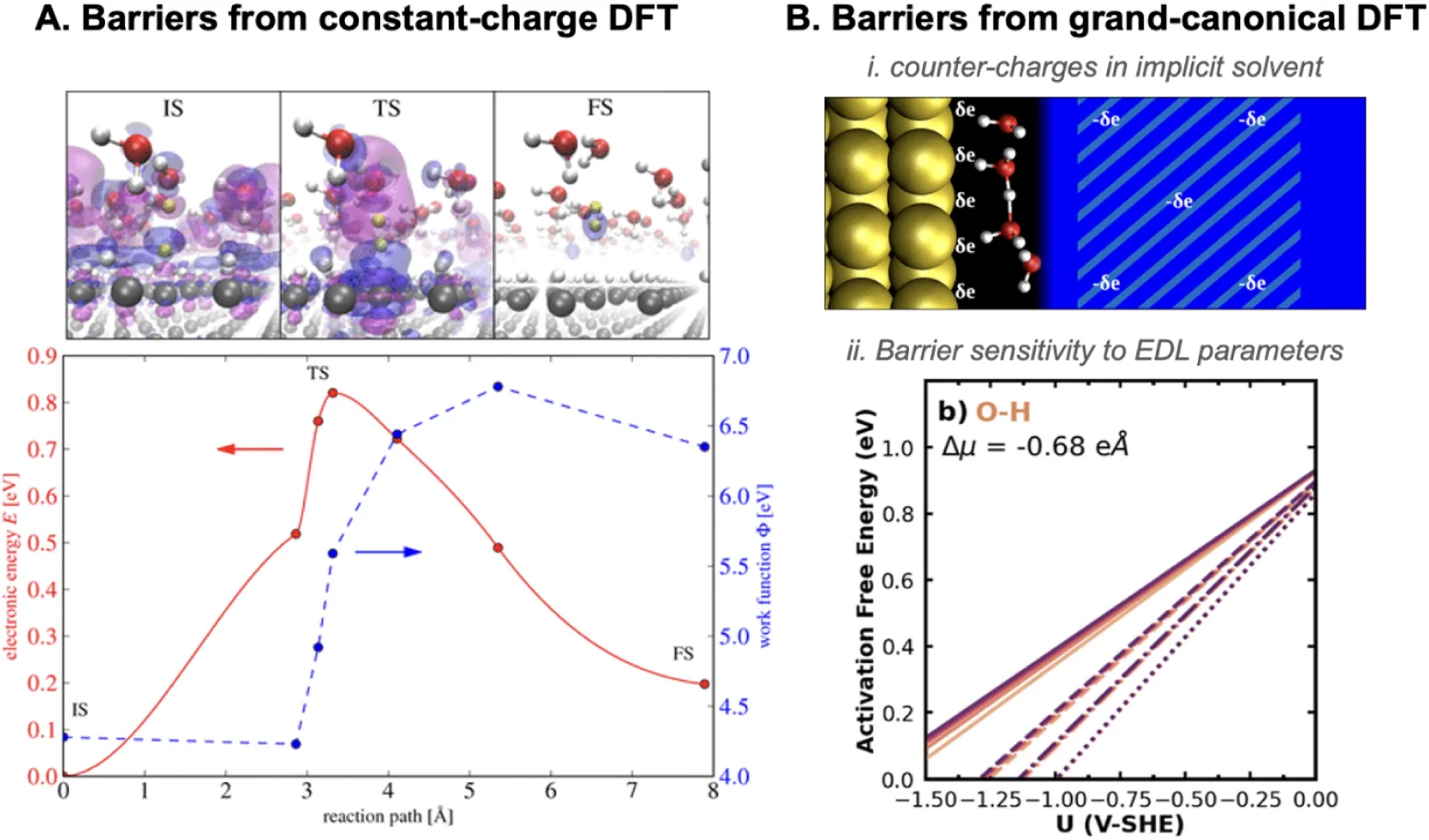
Activation energy calculations using constant-charge and grand-canonical DFT (GC-DFT) calculations. **A.** Constant-charge DFT calculation of a Heyrovsky reaction activation barrier, showing (top) the initial state (IS), transition state (TS), and final state (FS) structures, and (bottom) the electronic energy *E* and work function Φ along the reaction path. The work function (an analogue of the electrode potential) varies along the reaction coordinate of a constant-charge DFT calculation as charge is transferred between the solid and liquid phases to maintain zero total charge. Panel reproduced with permission from ref. 104. Copyright 2015 American Chemical Society. **B.** (*i*) Schematic view of a unit cell in a GC-DFT calculation where charges on solute are compensated by a countercharge distribution in implicit solvent. Panel reproduced with permission from ref. 107. Copyright 2018 American Chemical Society. (*ii*) Sensitivity of GC-DFT-calculated potential-dependent activation barriers for the CO* + H^+^ + e^−^ → COH* (O−H bond formation) step on Cu(111) to EDL parametrization. Different dielectric constants of 1, 2, 4, 8, 13, and 78 are shown by different colored lines, where darker lines correspond to larger values. Solid, dashed, dashed-dotted, and dotted lines correspond to EDL widths of 3, 4.5, 6, and 10 Å, respectively. Panel reproduced with permission from ref. 71. Copyright 2024 American Chemical Society.

Activation energies can be computed using either static or dynamic simulations. Static (i.e., 0 K) DFT calculations often use approaches such as the nudged elastic band method to identify transition state geometries on a ground-state potential energy surface that connects the reactant and product states of an elementary reaction. Such calculations can be performed using either implicit or explicit solvent, but this modeling approach entails a restricted sampling of the solvent microstates due to the absence of molecular kinetic energy. Furthermore, the structure of interfacial solvent and ions is sensitive to the applied potential. Barriers are sensitive to local solvation, and assuming a single arbitrary local solvent structure is unlikely to yield quantitatively accurate results. Alternately, enhanced sampling methods rooted in AIMD can be used to calculate free energies of activation via constrained simulations along a reaction coordinate defined by collective variables.^148–151^ Collective variables are often defined in terms of the lengths of the bonds formed or broken during the reaction; however, more complex definitions may be required to describe the free energy surface of electrochemical processes.^152^ Elementary steps in heterogeneous electrocatalysis are often proton-coupled electron transfer (PCET) reactions, which can occur when thermal fluctuation of the solvent to an activated state enables energy-conserving electron–proton tunneling.^17,153–159^ In such PCET reactions, the position of the transferring proton may not be a suitable reaction coordinate due to nuclear quantum effects. AIMD simulations have also been used to compute electron transfer barriers using the probability distribution of vertical energy gaps in equilibrated reactant and product trajectories.^160–166^ Related methods have recently been adapted to analyze interfacial electrochemical electron transfer in CO_2_ electroreduction.^167^ Rate constant models that account for quantum effects of transferring electrons and protons can provide further insights into the role of solvent dynamics on reactivity and kinetic isotope effects.^157,158,168–172^ Beyond transition-state theory, rate constant models based on Marcus–Hush–Chidsey or vibronically nonadiabatic PCET theory may be required to connect theoretical predictions to experimental data.^173–175^

The development of accurate first-principles rate constant models provides a necessary foundation for understanding kinetic reactivity in electrocatalysis. Yet there are many factors that influence the accuracy of calculated pre-exponential and exponential components of a rate constant. The approximations and limitations of these methods should be viewed as sources of error beyond the additional uncertainties stemming from the choice of DFT functional. These uncertainties are necessary to consider in a rigorous evaluation of electrochemical kinetics as DFT errors that may appear small translate to significant differences in calculated rate constants; e.g., transition-state theory rate constants change by a factor greater than 2000× for a DFT error of 0.2 eV at 298 K. Despite the complexities, assumptions, and uncertainties underlying theoretical activation barrier and rate constant calculations, we emphasize that the rate constant alone is not an experimental observable for a multistep catalytic reaction. *Thus, direct comparison of a computed barrier to an experiment is not an appropriate goal for a rigorous combination of theoretical and experimental electrocatalysis studies.* Still, there are opportunities to benchmark computational methods for accuracy in elementary rate constant predictions via comparisons to experimental measurements of well-defined active sites, where the kinetic currents associated with elementary electron transfer or PCET processes are separable from competing chemical and electrochemical reactions.^21,169,176–178^ Systematic theoretical studies may help elucidate trends in rate constants across catalysts and substrates via the establishment of Brønsted–Evans−Polanyi (BEP) relationships.^179,180^ The validity of linear free energy relationships suggests similar error cancellation across catalysts and can therefore enable trend-based comparisons. Deeper mechanistic insights can be obtained following integration of computed rate constants into microkinetic models, as will be discussed in the next section, where sensitivity can be analyzed and further experimental observables may be derived.

## Microkinetic Modeling and Kinetic Insights

Kinetic modeling provides a framework to link elementary reaction energetics and rate constants obtained from theory with experimentally observable macroscopic kinetics.^18,139,181–183^ Through this connection, quantities such as reaction rates (current densities), product selectivities (Faradaic efficiencies), apparent activation energies, reaction orders, and transfer coefficients can be evaluated and directly compared with experimental measurements. The values predicted by kinetic models, however, will inherit the error and imprecision of the underlying DFT-derived reaction energetics. To illustrate this, we examine the sensitivity of predicted macroscopic kinetic values to elementary step energies using a simple three step reaction involving: (1) equilibrated chemisorption of a reactant A, (2) an electrochemical irreversible proton-coupled electron transfer to form adsorbed AH*, and (3) an irreversible chemical step converting adsorbed AH* to products.

1 $$A+*↔A^{*},K=e^{-\Delta G_{\mathrm{ads}}/k_{B}T}$$

2 $$A^{*}+H^{+}+e^{-}\to AH^{*},k_{E}=Ae^{-E_{\mathrm{act}}^{E}/k_{B}T}e^{-\beta e\eta /k_{B}T}$$

3 $$AH^{*}\to \mathrm{products}+*,k_{C}=Ae^{-E_{\mathrm{act}}^{C}/k_{B}T}$$

In the above expressions, *A* is the pre-exponential factor, *T* is the temperature, *e* is the elementary charge, β is the symmetry factor for step 2, and η is the overpotential. The above equations use atomic units for energy terms (e.g., eV); however, the exponentials in eqs 1–3 can equivalently be written using energies in molar units (e.g., kJ mol^–1^) by replacing *k*_B_ with the gas constant *R* and *e* with the Faraday constant *F*. The equilibrium constant (*K*) depends on the reaction free energy for the chemisorption step Δ*G*_ads_, while the rate constants *k* depend on activation enthalpies *E*_act_. $E_{\mathrm{act}}^{E}$ is the electrochemical activation enthalpy for η = 0 in step 2, and $E_{\mathrm{act}}^{C}$ is the chemical activation enthalpy in step 3. The rate constant of the electrochemical step (*k*_E_) additionally depends on the applied η, unlike the rate constant of the chemical step (*k*_C_). Applying the pseudosteady-state hypothesis on AH* and employing the site balance, we derive the overall rate expression as

4 $$r=\frac{k_{E}K\left[A\right]\left[H^{+}\right]}{1+K\left[A\right]+\frac{k_{E}}{k_{C}}K\left[A\right]\left[H^{+}\right]}$$

Figure 4 illustrates the significant sensitivity of different kinetic observables with $E_{\mathrm{act}}^{E}$ while keeping all other values constant (including the chemical step activation energy of 0.6 eV). In this simple example, an expected 0.2 eV error in the electrochemical step activation barrier $\left(E_{\mathrm{act}}^{E}\right)$ at 0 overpotential between 0.5 to 0.7 eV can alter the apparent rate by at least 2 orders of magnitude and dramatically alter the steady-state surface coverage of reaction intermediates. Such wide changes in surface coverages would also impact the apparent reaction orders and transfer coefficients that are measured. The quantities plotted in Figure 4 are discussed in further detail in this section with context to integrated theoretical modeling and experimental measurements. Given the sensitivity of kinetic predictions to activation barriers along with challenges in using DFT to determine quantitatively accurate activation energies for electrochemical elementary steps, it is important to consider (and report) the sensitivity of predicted kinetic behavior to these values.

**Figure 4:**
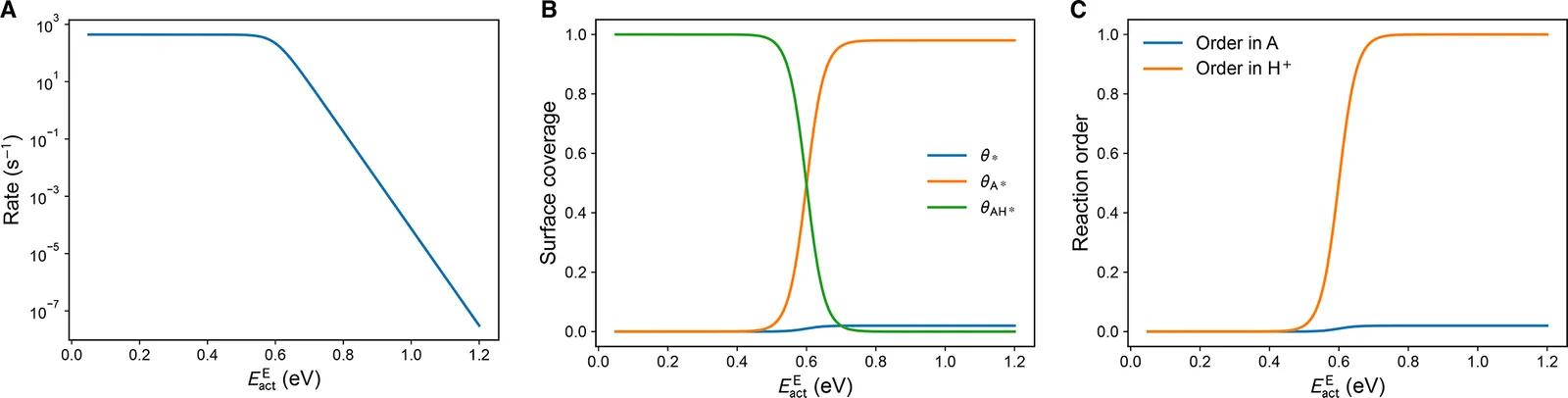
An illustrative example kinetic model. A simple kinetic model gives the **A**) rate, **B**) coverage of surface intermediates, and **C)** reaction orders. For the base three-step kinetic model, we used the following parameters *ΔG*_ads_ *=* −0.1 eV, *T* = 298 K, $E_{\mathrm{act}}^{C}=0.6\;\mathrm{eV},\beta =0.5,\eta =0,\left[A\right]=1,\left[H^{+}\right]=1,A$ = 6.2 × 10^12^ s^–1^.

Descriptor-based analysis is an approach to describe catalytic reactivity trends using a minimum number of thermodynamic variables within a parameter space governed by common mechanistic assumptions. The adsorption energy of one or two key reaction intermediates typically serve as descriptor(s); in turn, linear scaling relationships are used to obtain the adsorption energies of the other reaction intermediates,^184^ and BEP linear free energy relationships are used to obtain the activation energies.^185^ Relating adsorption and transition-state energies to descriptor(s) thus reduces the parameter space to describe reactivity trends using only one or two compact descriptors.^186^ These descriptor maps (or activity volcanoes) compare multiple catalysts through both experiments and simulations. When employing descriptors and scaling relations, DFT practitioners must consider quantitative total energy errors across density functionals and an expected error on absolute DFT energies of ∼0.1–0.2 eV.^187^ As shown in Figure 4, errors of this magnitude can propagate to order of magnitude differences in rate and qualitatively incorrect predictions of surface coverage, reaction orders, and transfer coefficients. Rigorous comparisons involving small energetic differences (e.g., 0.2 eV and below) are challenging given the propagation of error to computational quantities that can be directly compared with experiment. Descriptors provide more robust correlations when comparing energy differences over a range spanning an order of magnitude larger than this error, i.e., ∼2 eV. Scaling and BEP relationships are empirical correlations calibrated within a specific structural family, and their extrapolation to structurally distinct active sites is not guaranteed. Deviations from established scaling lines have themselves motivated design strategies for accessing reactivity outside the conventional descriptor space.^186^ Such extrapolative predictions should be validated with explicit DFT calculations rather than assumed to hold *a priori*. A representative example is for electrochemical CO_2_ reduction for which trends have been established among metal catalysts.^188^ In some cases, descriptor-based analysis lacking activation parameters can be analyzed in terms of the thermodynamic limiting potential;^41,189–191^ such assumptions must be validated in each case within the parameter space of interest. Developing meaningful descriptor-based analyses is difficult for reactions with limited working catalysts, such as electrochemical nitrogen reduction,^192^ or for reactions with hard requirements on stability, such as the oxygen evolution reaction.^193^ More rigorous kinetic analyses are needed in these scenarios to develop quantitative connections to experimental observables beyond limiting potentials and reaction rates (current densities).

Microkinetic models (MKM) possess both explanatory and predictive capabilities, allowing catalysis researchers to interpret and rationalize experimental kinetics from a specific catalytic material or trends across catalytic materials. This provides valuable insights into the mechanisms, active sites, and kinetic parameters that characterize the elementary steps of the reaction. In contrast to the simplified rate expression in eq 4, MKMs do not require simplified assumptions about quasi-equilibrium, reversibility, or steady state. Additionally, microkinetic models can be utilized in conjunction with descriptor-based tools such as scaling relations and BEP relationships to extend beyond observed materials and predict candidates for synthesis and testing of their catalytic activity.^185,186^ MKMs vary in complexity from mean-field approaches, which describe catalytic surfaces through spatially averaged properties, to kinetic Monte Carlo (KMC) simulations that explicitly resolve individual surface events-adsorption, diffusion, reaction, and desorption-on discrete lattice sites. KMC methods treat each elementary step probabilistically according to its rate constant, yielding a stochastic representation of surface dynamics. The commonly implemented mean-field models offer computational efficiency and analytical tractability for extracting kinetic parameters yet neglect spatial correlations and local coverage heterogeneities that KMC naturally captures at the atomistic scale. Comparing the kinetic insights from the model (e.g., reaction orders, Tafel slopes, apparent activation energies) with experimental kinetic observations provides independent points of contact, transforming microkinetic models from black-box rate predictors into falsifiable, multiconstraint diagnostic tools. This Perspective argues that rigorous theoretical electrocatalysis demands systematic validation of microkinetic models against this full suite of observables, and that the nature of active site structures, coverages, and activation energy barriers predicted by these models must feedback consistently into the DFT calculations used to parametrize them.^203^

*Steady-state adsorbate* coverages represent perhaps the most fundamental output of microkinetic models, yet they remain among the most underutilized for model validation purposes. Coverage predictions matter for multiple reasons. First, they provide direct comparison points with *operando* spectroscopic techniques. Surface-enhanced infrared absorption spectroscopy (SEIRAS), ambient-pressure X-ray photoelectron spectroscopy (APXPS), and surface-enhanced Raman spectroscopy (SERS) can identify which intermediates populate electrode surfaces under working conditions and, increasingly, provide quantitative or qualitative coverage estimates.^10^ Second, surface coverages can significantly modify the adsorption energies and activation energies, thus changing the reaction rates and selectivities.

The *reaction order* with respect to each species—defined as n_i_ = (∂ln­(r)/∂ln­(C_i_))—provides a mechanistic fingerprint that depends sensitively on which steps are rate-limiting and which species participate in quasi-equilibrated presteps. Similarly, pH dependence (effectively the reaction order in H^+^ or OH^–^) diagnoses whether proton-coupled electron transfer steps limit rates.^197^ We encourage experimentalists to determine *reaction orders*, while being cautious to avoid confounding effects from mass transport and change in double-layer structure.^18^ Theoretical studies predicting *reaction rates* rarely calculate and compare predicted reaction orders against experimental values. This omission is particularly troubling because reaction orders provide model discrimination power orthogonal to absolute rates. Two different mechanisms might predict similar current densities at one set of conditions but divergent behavior when reactant concentrations vary. A model correctly predicting rates but incorrectly predicting reaction orders has likely achieved the right answer for the wrong reasons—perhaps through compensating errors in adsorption energies and barriers.

*Tafel slope*—the derivative of overpotential with respect to log current density—encodes information about the number of electrons transferred in, and prior to, kinetically relevant step(s).^142,204^ Note that Tafel slopes can be alternatively expressed in terms of the *transfer coefficient*, a nondimensional quantity that is proportional to the reciprocal of the Tafel slope and quantifies the rate–potential scaling of a reaction. Standard microkinetic models in electrocatalysis incorporate potential dependence through the symmetric Butler–Volmer approximation or more sophisticated models of the electrochemical double layer. These models predict how Tafel slopes should vary with potential, often showing transitions between different kinetic regimes as coverage changes or different steps become rate-limiting. The experimental literature on electrocatalysis routinely reports Tafel slopes, making this one of the most accessible validation metrics. Experimentally, Tafel slopes can be obtained from voltammetry, chronoamperometry and chronopotentiometry, or impedance, but different techniques may suffer from a convolution of kinetic and nonkinetic (e.g., mass transport) contributions to the measured Tafel slope. Considering the differential character of Tafel slopes by analyzing its dependence on current and/or potential enables experimentalists to disentangle kinetic and nonkinetic effects, and to develop understanding of reaction mechanisms.^139,205,206^ Despite the rich mechanistic information encoded by Tafel slopes and complementary apparent quantities (e.g., reaction orders),^142,207^ theoretical studies commonly calculate current–voltage curves without extracting and comparing Tafel slopes, or worse, report only fitted Tafel slopes from narrow potential windows without discussing whether predicted slopes match experimental values or whether the model predicts slope transitions at specific potentials. Theoretical studies can add mechanistic insight into experimentally measured Tafel slopes because mechanistic interpretation based on “cardinal” values can be tenuous, especially far from equilibrium.^142,208,209^

Perhaps the most critical requirement for rigorous theoretical microkinetic modeling is consistency between predicted coverages and the DFT calculations used to parametrize the model.^182^ Standard computational workflows calculate adsorption energies and activation energies on low-coverage or clean surfaces (typically 1/9 to 1/4 ML in periodic slab calculations) using well-defined surface structures. However, if the resulting microkinetic model predicts high adsorbate coverages (>0.5 ML) or mixed adsorbate layers under operating conditions, the energetics calculated at low coverage may be severely incorrect due to through-space dipole coupling, through-surface electronic effects, and direct steric repulsion that can shift adsorption energies by 0.2–0.5 eV or more as coverage increases.^194,198^ Moreover, activation energies for elementary steps involving adsorbates also exhibit coverage dependence, with transition states destabilized by crowding or stabilized by cooperative effects with spectator species. Ignoring these effects when constructing microkinetic models is equivalent to assuming the energetics remain constant regardless of surface composition—an assumption virtually never satisfied at the high coverages typical of electrocatalytic operating conditions and can result in drastically different kinetic parameters compared to experimental observables.^18,46,49^ Therefore, we recommend a *coverage-energetics feedback loop* for building theoretical microkinetic models that starts from low coverage and gradually increases the complexity of the model, i.e., incorporating coverage dependent adsorption and activation energies, while comparing the kinetic observables from the model (reaction orders, Tafel slopes, apparent activation energies, etc.) with those obtained from experimental kinetic measurements.^5,182^ Recent microkinetic studies demonstrate that this vision is achievable.^18,86,194–202^

## Recommendations

Despite the challenges in modeling the complex chemistry at the electrocatalytic interface, there are various opportunities to obtain valuable mechanistic insights through the integration of theory and experiment. The above discussions and examples describe useful strategies and opportunities to improve connections in collaborative theoretical and experimental electrocatalysis research. Some inputs and outputs of theoretical models may be directly compared to experimentally measured quantities (e.g., adsorption energies), whereas some quantities often cannot be directly measured for multistep reactions (e.g., elementary rate constants and intrinsic activation barriers). Uncertainties in first-principles models for the effects of applied potential and the response of the electric double layer complicate the calculation of adsorption energies at solid–liquid interfaces; however, computational models can be calibrated alongside various experimental techniques (e.g., voltammetry, mass spectrometry, calorimetry). First-principles rate constant predictions suffer from similar sources of error and uncertainty, but comparisons to well-defined and separable elementary processes such as hydrogen underpotential deposition could enable rigorous benchmarking for functionals and solvation treatments. Uncertainties and inherent error underlying DFT modeling in electrocatalysts propagate to first-principles predictions of macroscopic kinetics; however, kinetic observables such as apparent activation energies, reaction orders, and transfer coefficients that are obtained from derivatives of reaction rates can elucidate important mechanistic information such as rate-controlling steps and steady-state surface coverage. We encourage theoretical and experimental practitioners to establish points of connection via such kinetic observables that can benefit from systematic error cancelation more than comparisons of absolute rates.

There are many well-established approaches and examples in the thermal heterogeneous catalysis that should inspire future theory and experiment integration efforts in heterogeneous electrocatalysis.^181,182,210,211^ The thermal catalysis literature is rich with many well-designed experiments and simulations that have been used to understand catalyst structure, adsorption energetics, and reaction kinetics. In the absence of an applied potential, experimental databases have been established as benchmarks for electronic structure theory to quantify adsorption energies,^75,212,213^ activation energies,^214^ and solvent adhesion energies^125,126^ required to evaluate equilibrium and rate constants of elementary steps in thermal catalysis.^215^ In the absence of experimental data to quantify energetics elementary steps, microkinetic modeling can be used in conjunction with spectroscopy, as well as experimentally measured reaction orders and apparent activation energies, to bridge insights from theory and experiment.^181,182^ The theoretical and experimental tools established for fundamental thermal heterogeneous catalysis research should be adapted to electrocatalysis research to establish rigorous mechanistic interpretations. Yet theorists and experimentalists alike should both consider the additional challenges and uncertainties associated with applying these tools to the study of solid–liquid electrocatalytic interfaces.

Theorists should consider model uncertainties and inherent errors when attempting to relate calculated quantities to experimental measurement. Over the past few decades, DFT usage has grown in catalysis research and has become an indispensable tool for researchers aiming to understand the molecular origins of catalytic behavior. The entry barrier for using DFT in catalysis research has also been lowered, concomitant with a userbase that has expanded beyond the domain of electronic structure theorists. There are clear upsides to improved accessibility and availability of DFT to the catalysis community, as it enables more researchers to develop atomistic models to understand experimental measurements and predict trends in catalyst properties. Yet the increased availability of these tools does not avoid sources of error in the chosen catalyst model, electronic structure theory, rate constant model or proposed mechanism. Furthermore, each possible source of error ultimately propagates to first-principles predictions of catalytic rates. DFT should not be used as a crutch in the absence of more rigorous experiments, as modeling choices can qualitatively or quantitatively be tuned to align with experimental trends. Researchers should resist the urge to employ such uses of “decorative DFT” to obtain a desired match with experiment. Instead, we encourage theorists and experimentalists to collaborate iteratively to establish as many connection points as possible to obtain more robust and comprehensive conclusions about the catalytic system being studied, and when applicable, aim to establish principles that are transferrable beyond the specific catalytic system. Where available, experimental characterization should be leveraged to rationalize models of the catalyst under reaction conditions. In the absence of such experimental insights, a theoretical finding that remains robust across a diverse family of active site structures can be more valuable than quantitative agreement for one idealized structure, as exemplified by trend- and descriptor-based approaches that identify principles conserved across structural families.

Experimentalists should aim to provide theorists with multiple points of comparison to self-consistently improve computational models and molecular-level understanding of experimental measurements. Experimentalists should acknowledge where each catalyst lies on a spectrum from well-defined (e.g., single crystals) to ill-defined (e.g., amorphous oxide) and select appropriate tools to characterize their structures, recognizing the limitations in each case. Surface science studies play an important role in defining surface structures that can be matched by theory,^27–29^ although technical catalysts may show distinct performance. Experimentalists should characterize the composition and structure of electrocatalysts *operando* and should acknowledge that significant structural changes can occur under reaction conditions if *ex situ* characterization alone is performed. Experimentalists should ensure that activity measurements are free of artifacts that could undermine integration between experiment and theory.^18^ Experimentalists should aim to establish multiple points of contact with theory by supplementing activity data with measurements of reaction orders, pH dependence, Tafel slopes, adsorbate identities, etc. Finally, experimentalists should aim to understand the limitations of the theoretical methods being used by their collaborators so that they can develop questions appropriate for the level of theory being employed and so that theoretical insights are not overstated.

## Outlook

We have outlined considerations and recommendations for researchers aiming to integrate theory and experiment for rigorous mechanistic studies in electrocatalysis. There are many tools that theoretical and experimental electrocatalysis researchers can leverage to obtain mechanistic insights into electrocatalytic reactivity that are deeper than the sum of the two parts. Rigor and reproducibility are enhanced when theory and experiment intersect via multiple points of connection in the study of any given system. This is not to say that everything above must be satisfied for a particular study to be insightful. We contend that there remains great value in solely theoretical or experimental electrocatalysis research. Developments in theory are needed to develop robust models of electric double layers or electron transfer rate constants, as well as to propose descriptor-based reactivity predictions and explore the feasibility of catalytic reactivity at a proposed active site. The combination of theory and experiment is needed to address knowledge gaps that cannot be filled by one approach alone. Taken together, this work provides strategies for an integrated—rather than decorative—combination of theory and experiment in electrocatalysis research.
